# Nutraceutical Eriocitrin (Eriomin) Reduces Hyperglycemia by Increasing Glucagon-Like Peptide 1 and Downregulates Systemic Inflammation: A Crossover-Randomized Clinical Trial

**DOI:** 10.1089/jmf.2021.0181

**Published:** 2022-11-09

**Authors:** Thais Borges Cesar, Fernanda Maria Manzini Ramos, Carolina Barbosa Ribeiro

**Affiliations:** Laboratory of Nutrition, School of Pharmaceutical Sciences, Sao Paulo State University (UNESP), Araraquara, São Paulo, Brazil.

**Keywords:** citrus flavonoids, eriocitrin, GLP-1, hyperglycemia, nutraceutical, systemic inflammation

## Abstract

This double-blind, randomized, placebo/controlled, crossover study evaluated the efficacy of Eriomin^®^ in reducing hyperglycemia and improving diabetes-related biomarkers in individuals with hyperglycemia above 110 mg/dL (mean 123 ± 18 mg/dL). Subjects (*n* = 30), divided into two groups (Eriomin or Placebo), who received a dose of 200 mg/d of the designated supplement for 12 weeks and, after a washout period of 2 weeks, switched to the other supplement in the following 12 weeks. Assessments of biochemical, metabolic, inflammatory, blood pressure, anthropometry, and dietary parameters were performed at the beginning and end of each intervention. Treatment with 200 mg/d of Eriomin significantly decreased blood glucose (−5%), homeostasis model assessment of insulin resistance (−11%), glucagon (−13%), interleukin-6 (−14%), tumor necrosis factor alpha (−20%), and alkaline phosphatase (−13%); but increased glucagon-like peptide 1 (GLP-1) by (17%) (*P* ≤ .05). At the end of the placebo period, there was a 13% increase in triglycerides (*P* ≤ .05). Other parameters evaluated did not change with Eriomin or placebo. In conclusion, intervention with Eriomin benefited the glycemic control of prediabetic and diabetic patients, with higher blood glucose levels, by increasing GLP-1 and decreasing systemic inflammation.

## INTRODUCTION

Currently, type 2 diabetes (T2D) affects almost a half billion people worldwide (468 million in 2017), corresponding to 6.3% of the world population, with the majority being individuals over 50 years of age and, to a lesser extent, adolescents and young adults who already show signs of the disease.^1^ The risk of developing diabetes 2 is determined by multiple genetic loci and their interactions with lifestyle and environmental factors.^2^ A sedentary lifestyle, poor eating habits, with high consumption of ultra-processed/processed and high-energy foods, excess visceral fat, overweight, and obesity can lead to metabolic complications that contribute to T2D onset. Thus, it is essential to implement health treatments in the early stages of the disease, before the beginning of complications.^3^

This period with initial metabolic/biochemical alterations, known as prediabetes, is recognized by signs such as altered fasting glycemia, impaired glucose tolerance, or the combination of both previous conditions; and glycated hemoglobin (HbA1c) between 5.7% and 6.4%.^4^ However, a reduction in hyperglycemia and other additional metabolic factors in this critical period has a strong chance of reversing this condition, before the definitive outbreak of the disease.^5^

Although traditional drug treatments, such as metformin, are effective in reducing hyperglycemia, they can lead to a series of side effects limiting the chronic use by sensitive patients.^6^ Thus, current research has sought alternative treatments that can increase insulin secretion and maintain blood glucose homeostasis through new therapeutic approaches. Polyphenolic compounds emerged in this research segment as nutraceuticals and hypoglycemic agents, which apparently have little or no side effects with acute and chronic use. They potentially can reduce postprandial hyperglycemia by inhibiting glucose uptake and its release from the intestine to the liver, stimulating insulin secretion by the pancreas, and increasing glucose uptake by muscles and adipocytes.^7^

These events occur through the direct action of polyphenols in the intestinal cells, stimulating the production of the hormone incretin glucagon-like peptide-1 (GLP-1), which in turn increases insulin secretion, and also through an indirect action on the microbiota in the distal portion of the intestine. The transformation of polyphenols by resident gut bacteria increases their bioavailability and the production of short-chain fatty acids, which will have favorable metabolic effects to reduce hepatic glucose secretion. Polyphenols are also involved in the regulation of microRNAs associated with glycemic control.^7^ Among them, quercetin, genistein, and citrus flavonoids, such as hesperidin and eriocitrin, have been studied.^8–13^

A previous randomized clinical study has shown that regular supplementation with a mix of eriocitrin plus other citrus flavonoids (Eriomin^®^) in mice reduces fasting glucose and other inflammatory biomarkers related to development of diabetes.^12^ In contrast, levels of GLP-1 and antioxidant capacity in the blood circulation were also increased. In addition, Eriomin was able to significantly reduce diabetes-related parameters in prediabetic patients, such as fasting hyperglycemia (−5%), insulin resistance (−7%), glucose intolerance (−7%), and glycated hemoglobin, reversing 24% of these patients to normal glycemia status. Based on these positive results, we proposed to evaluate the effect of supplementation with Eriomin for 12 weeks in prediabetic with higher levels of hyperglycemia, over 110 mg/dL, without complications and/or use of hypoglycemic agents. The hypothesis of this study is that Eriomin can reduce hyperglycemia and inflammatory biomarkers in moderate to high hyperglycemia prediabetic and diabetic patients without hypoglycemic agents.

## MATERIALS AND METHODS

### Individuals

Individuals aged 40–70 years with increased fasting glycemia (110–150 mg/dL or 6.1–8.6 mmol/L) were eligible to participate in this study. Exclusion criteria were based on physiological conditions or chronic diseases that interfere with blood glucose levels, such as use of hypoglycemic or weight loss drugs, dietary supplements (vitamins, minerals, bioflavonoids, probiotics, symbiotic, or other bioactive compounds), and intense physical exercise (>10 h/week).

**FIG. 1.**
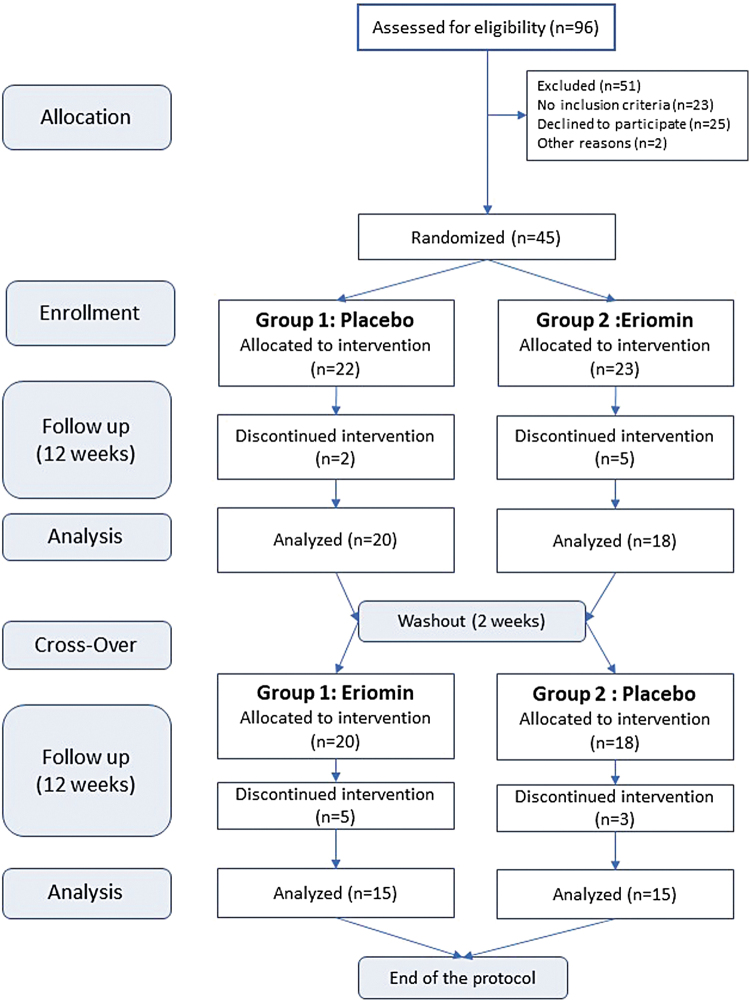
Experimental design of a 26-week, double-blind, randomized, placebo-controlled, crossover study. From 96 candidates, 45 individuals were selected and randomly divided into two groups: Placebo (*n* = 22) and Eriomin (*n* = 23). For 12 weeks, they received a daily dose of the designated supplement, and then switched to the other supplement for the next 12 weeks. All underwent a 2-week washout period between treatments.

A double-blind, randomized, placebo/controlled, and crossover study for 26 weeks was performed. From 96 applicants, 45 individuals selected were randomly divided in two groups: Placebo (*n* = 22) and Eriomin (*n* = 23). For 12 weeks, they received a daily dose of supplement, according to the group, they were assigned, and then switched to another type of supplement for the next 12 weeks with a 2-week washout period between the treatments (Fig. 1). The washout period was estimated to be equivalent to five or more times the period of the bioflavonoid half-life in the blood.

All subjects and the principal investigator remained blind to the treatments until all analyses are completed by an additional researcher not directly involved in the study as a participant or in the execution of the dosing program.^*^ This protocol was approved by the Ethical Board of the Pharmacy School of Sao Paulo State University and registered at ClinicalTrials.gov (NCT03928249). All individuals signed an Informed Consent Form before starting the study.

### Supplements preparation

The intervention product was Eriomin, provided by Ingredients by Nature™ (Montclair, CA, USA), which composition is 70% eriocitrin, 5% hesperidin, 4% naringin, 1% didymin, and 20% of fiber material, from epidermis and periderm cell-wall, such as suberin, cutin, lignin, pectin, and cellulose. The dose of 200 mg of active ingredients per capsule is equal to 140 mg of eriocitrin plus 10 mg of hesperidin plus 8 mg of naringin plus 2 mg of didymin. This dose was chosen after the previous clinical study showed that the lower dose of 200 mg had an effect comparable to the higher doses of 400 and 800 mg for the main biochemical and molecular parameters studied, and better efficacy on hypoglycemic action.^12^

The placebo was composed of microcrystalline corn starch. Placebo and Eriomin were encapsulated in tablets of the same size, shape, and color, by a registered pharmacist. Volunteers were instructed to consume one capsule after dinner with a glass of water for 24 weeks, with 2 weeks of washout without supplement between the two 12-week cycles. Supplements were given to participants every 2 weeks after randomization, and they were asked to bring back the flasks to the next appointment (every 2 weeks) with all capsules that had left.

### Anthropometric assessment and dietary intake

In the beginning of the first, 12, 14, and 26 weeks, the volunteers were evaluated for the following anthropometric parameters: body weight (kg), lean mass (kg), and fat mass using InBody 720 (Biospace, Tokyo, Japan). Arterial blood pressure was measured with digital monitor ReliOn (HEM-741 CRELN, USA). Subjects were instructed to maintain their usual diet and physical activity during the total experimental period.

Volunteers were registered for their consumption of all foods and beverages in a Food Record form at regular intervals during the experimental period. Data analysis of energy, macronutrients, and micronutrients intake was performed through the DietBox^®^ software, based on the Brazilian Table of Food Composition.^14^

### Biochemical measurement

Overnight fasting blood samples were obtained in the 1st, 12th, 14th, and 26th week of intervention at the São Lucas Clinical Analyzes Laboratory, Araraquara-SP, and blood serum were stored at −80°C. Fasting and postprandial glucose, HbA1c, total cholesterol, high-density lipoprotein-cholesterol (HDL-C), triglycerides, aspartate transaminase (AST), alanine transaminase (ALT), alkaline phosphatase (ALP), gamma-glutamyl transferase (*γ*GT), and uric acid and creatinine were performed by commercial kits (Labtest, Brazil). Homeostasis model assessment of insulin resistance (HOMA-IR) was calculated, and the cutoff set was at ≥2.71. Lipid peroxidation was assessed by thiobarbituric acid reactive substances (TBARS) assay and total antioxidant capacity by radical 2,2′-azino-bis(3-ethylbenzothiazoline-6-sulphonic acid) (ABTS) + assay. Tumor necrosis factor alpha (TNF-*α*), interleukin 6 (IL-6), and high-sensitivity C-reactive protein (hsCRP) were performed by Luminex Milliplex^®^ (RP3X Scientific, Ribeirao, SP, Brazil).

### Compliance assessment

During the intervention, the patients were asked about any discomfort and adverse events. The conformity of the volunteers was assessed by counting the capsules not taken during each experimental period.

### Primary and secondary outcome measures

The primary outcome was fasting blood serum glucose. The secondary outcomes were insulin, HOMA-IR, HbA1c, GLP-1, Glucagon, total cholesterol, triglycerides, HDL-C, low-density lipoprotein-cholesterol (LDL-C), ALP, AST, ALT, *γ*GT, TNF-*α*, IL-6, hsCRP, antioxidant capacity and serum lipid peroxidation, body weight, body mass index (BMI), muscle mass, fat mass, and systolic and diastolic blood pressure

### Statistical analysis

Sample size was established using the SigmaStat32 software, with significance level of 5% and 80% power. The calculation of the sample size was based on the results of our previous experiment with Eriomin.^12^ Considering that the reduction of glycemia was the main outcome expected in this study, this variable was used to calculate the sample size. Thus, the estimated minimum sample size, considering a dropout rate of ∼15%, was 20 individuals per group.

Data are presented as mean ± standard deviation. Statistical analysis was performed using SPSS 22 (Statistical Package Social Sciences). One-way analysis of variance (ANOVA) was used to identify differences between groups in the baseline period. Two-way repeated measures ANOVA followed by Sidak *post hoc* test was applied to compare changes within and between groups, and the statistical significance is *P* ≤ .05.

## RESULTS

### Individuals

Ninety-six individuals, previously reported as hyperglycemic, signed up to participate in this study. Among them, 51 participants were excluded for the following reasons: 23 did not meet the trial criteria; 25 refused to continue participating in the experiment due to COVID-19 restrictions and, 2 for personal reasons (Fig. 1). Of these, 45 remaining subjects were selected and randomly assigned to the two trial groups: Placebo and Eriomin. The baseline characteristics of these participants were similar between groups, as shown in Table 1.

**Table 1.** Baseline Characteristics of Prediabetic + Diabetic Volunteers Submitted to Eriomin (200 mg/d) or Placebo Supplementation for 12 Weeks

	Female	Male	Total
Variables	*N* = 11	*N* = 19	*N* = 30
Age, years	50.3 ± 11.2	52.4 ± 9.7	51.6 ± 10.3
Glucose, mg/dL	123 ± 11	122 ± 12	123 ± 18
Insulin, *μ*U/mL	29.3 ± 20.1	19.7 ± 10.6	23.3 ± 15.3
HbA1c, %	6.53 ± 0.49	6.29 ± 0.83	6.38 ± 0.72
Total cholesterol, mg/dL	187 ± 32	189 ± 47	189 ± 42
Triglycerides, mg/dL	154 ± 81	170 ± 81	164 ± 80
Weight, kg	96.4 ± 17.2	106 ± 30	102 ± 26
BMI, kg/m^2^	35.5 ± 5.2	34.6 ± 8.1	34.9 ± 7.1
Waist circumference, cm	109 ± 9	111 ± 16	110 ± 14
Systolic blood pressure, mmHg	119 ± 10	122 ± 13	121 ± 12
Diastolic blood pressure, mmHg	75.5 ± 8.2	79.3 ± 11.6	76.0 ± 10.4

Data are presented as mean ± SD.

BMI, body mass index; HbA1c, glycated hemoglobin; SD, standard deviation.

### Biochemical markers

Fasting blood glucose levels were reduced after Eriomin treatment. A reduction of 5% was observed at a dose of Eriomin of 200 mg (*P* ≤ .02) at the end of treatment. In contrast, subjects in the placebo group had a nonsignificant increase in fasting blood glucose levels after 12 weeks (Table 2). In addition, there was a nonsignificant reduction of −2% in HbA1c levels after treatment with Eriomin (*P* ≥ .05) (Table 2). All volunteers had insulin resistance at the beginning, during, and at the end of the experiment (HOMA-IR ≥2.71), but there was a reduction of 11% (*P* ≥ .05) after intervention with 200 mg of Eriomin (Table 2). Regarding blood lipids, there was no significant reduction in total cholesterol, LDL-C, HDL-C, but triglycerides were increased in both groups at the end of the experimental period, with 13% of significance difference for Placebo (*P* ≤ .02) (Table 2).

**Table 2.** Blood Serum Biochemical Markers of Prediabetic + Diabetic Volunteers Submitted to Eriomin (200 mg/d) or Placebo Supplementation for 12 Weeks

Variables		Placebo		Eriomin	
		0 Week	12° Week	0 Week	12° Week
Glucose, mg/dL		117 ± 14	122 ± 20	122 ± 17^a^	116 ± 15^b^
		*4%*		*−5%* ^*^	
Insulin, *μ*U/mL		19.4 ± 9.4	19.0 ± 9.4	23.1 ± 14.9	22.2 ± 12.5
		*−2%*		*−4%*	
HOMA-IR		5.63 ± 2.86	5.89 ± 3.28	7.36 ± 4.32	6.54 ± 4.11
		*5%*		*−11%*	
HbA1c, %		6.27 ± 0.63	6.31 ± 0.62	6.36 ± 0.74	6.23 ± 0.63
		*0.6%*		*−2.1%*	
Total cholesterol, mg/dL		187 ± 42	189 ± 38	183 ± 33	184 ± 39
		*1%*		*0.7%*	
LDL-C, mg/dL		111 ± 33	109 ± 33	109 ± 28	107 ± 32
		*−2.0%*		*−2.1%*	
HDL-C, mg/dL		43.2 ± 12.5	43.8 ± 12.1	43.0 ± 9.5	43.2 ± 12.8
		*1%*		*0.5%*	
Triglycerides, mg/dL		160 ± 79^a^	180 ± 91^b^	149 ± 67	163 ± 73
		13%^*^		*10%*	

Two-way repeated measures ANOVA followed by Turkey test between groups (Placebo vs. Eriomin) over 12-wk intervention period.

(^a,b^)different superscript lowercase letters indicate statistical difference within the group (*P* ≤ 0.05).

*Italic values:* difference in percentage between week 12 and week 0.

(^*^) means statistical difference between week 0 and 12 (*P* ≤ 0.05).

ANOVA, analysis of variance; HDL-C, high-density lipoprotein-cholesterol; HOMA-IR, homeostasis model assessment of insulin resistance; LDL-C, low-density lipoprotein-cholesterol.

### Metabolic and inflammatory markers

Levels of blood plasma GLP-1 increased significantly 17% for Eriomin group (*P* < .04). After intervention, Eriomin supplementation also promoted an average reduction of −13% of glucagon (*P* < .001), which was statistically different from the end point of Placebo. This group showed no difference between from the beginning to the final measurement of glucagon levels (Table 3).

**Table 3.** Metabolic and Inflammatory Blood Markers of Prediabetic Volunteers Submitted to Eriomin (200 mg/d) or Placebo for 12 Weeks

Variables		Placebo		Eriomin	
		0 Week	12° Week	0 Week	12° Week
GLP-1, rmol/L		23.4 ± 19.8	22.6 ± 18.2^A^	28.3 ± 21.3^a^	33.0 ± 22.8^b,B^
		*−3%*		*17%* ^*^	
Glucagon, pg/mL		80.7 ± 50.5	82.2 ± 49.3^A^	77.9 ± 50.4^a^	67.5 ± 42.3^b,B^
		*2%*		*−13%* ^*^	
hsCRP, mg/dL		0.46 ± 0.44	0.43 ± 0.40	0.45 ± 0.40	0.42 ± 0.40
		*−7%*		*−5%*	
IL-6, pg/mL		10.7 ± 5.0	10.1 ± 5.0	12.1 ± 6.7^a^	10.5 ± 6.4^b^
		*−6%*		*−14%* ^*^	
TNF-*α*, pg/mL		5.22 ± 2.24	4.96 ± 2.57	6.15 ± 3.21^a^	4.93 ± 2.08^b^
		*−5%*		*−20%* ^*^	
Antioxidant capacity, *μ*M		0.68 ± 0.73	0.69 ± 0.74	0.69 ± 0.75	0.69 ± 0.75
		*1%*		*0%*	
Lipid peroxidation, (MDA) mM		6.81 ± 3.87	8.23 ± 6.54	7.06 ± 4.01	7.83 ± 5.84
		*21%*		*11%*	

Two-way repeated measures ANOVA followed by Turkey test between groups (Placebo vs. Eriomin) over 12-wk intervention period.

(^a,b^)different superscript lowercase letters indicate statistical difference within the group (*P* ≤ 0.05).

(^A,B^)different superscript uppercase letters indicate statistical difference inter-groups (*P* ≤ 0.05).

*Italic values:* difference in percentage between week 12 and week 0.

(^*^) means statistical difference between week 0 and 12 (*P* ≤ 0.05).

GLP-1, glucagon-like peptide 1; hsCRP, high-sensitivity C-reactive protein; IL-6, interleukin 6; MDA, malondialdehyde; TNF-*α*, tumor necrosis factor alpha.

Eriomin supplementation promoted a mean reduction of 14% in IL-6 (*P* ≤ .05) and 20% in TNF-*α* (*P* ≤ .05) after 12 weeks. Other tested variables, such as hsCRP, antioxidant capacity, and malondialdehyde marker for lipid peroxidation, did not show differences between the beginning and the end of the experiment for both Placebo and Eriomin group (Table 3).

Hepatic enzymes (*γ*GT, AST, ALT) and markers of kidney function (urea and creatinine) remained unchanged during the experiment in groups supplemented with Eriomin or Placebo (Table 4). However, ALP decreased 8% (*P* = .02) after Eriomin treatment, but not after placebo (Table 4).

**Table 4.** Liver and Kidneys Function Markers of Prediabetic Volunteers Submitted to Eriomin (200 mg/d) or Placebo for 12 Weeks

Variables		Placebo		Eriomin	
		0 Week	12° Week	0 Week	12° Week
ALP, U/L		58.1 ± 14.3	59.2 ± 15.7^A^	57.6 ± 16.7^a^	53.1 ± 13.6^b,B^
		*2%*		*−8%* ^*^	
*γ*GT, U/L		46.9 ± 32.1	46.4 ± 28.9	37.4 ± 21.1	39.1 ± 22.1
		*−1%*		*3%*	
AST, U/L		22.4 ± 7.9	22.1 ± 6.6	21.1 ± 7.1	22.3 ± 7.7
		*−1%*		*6%*	
ALT, U/L		28.1 ± 15.3	28.1 ± 13.4	26.7 ± 15.0	27.4 ± 13.3
		*−0.5%*		*3%*	
Urea, mg/dL		32.0 ± 9.9	31.4 ± 9.7	33.7 ± 12.0	33.4 ± 10.2
		*2%*		*−1%*	
Creatinine, mg/dL		0.85 ± 0.24	0.86 ± 0.22	0.87 ± 0.26	0.86 ± 0.22
		*2%*		*−2%*	

Two-way repeated measures ANOVA followed by Turkey test between groups (Placebo vs. Eriomin) over 12-wk intervention period.

(^a,b^)different superscript lowercase letters indicate statistical difference within the group (*P* ≤ 0.05).

(^A,B^)different superscript uppercase letters indicate statistical difference inter-groups (*P* ≤ 0.05).

*Italic values:* difference in percentage between week 12 and week 0.

(^*^) means statistical difference between week 0 and 12 (*P* ≤ 0.05).

*γ*GT, gamma-glutamyl transferase; ALP, alkaline phosphatase; ALT, alanine transaminase; AST, aspartate transaminase.

### Hemodynamic, anthropometry, and dietary variables

Supplementation with Eriomin had no effect on blood pressure (systolic and diastolic), body weight, BMI, lean mass, fat mass, and ratio hip/waist (Table 5). Also, no effect of Eriomin was observed on dietary parameters after 12 weeks of either treatment (Table 6).

**Table 5.** Hemodynamic and Anthropometric Parameters of Prediabetic Volunteers Submitted to Eriomin (200 mg/d) or Placebo for 12 Weeks

Variables		Placebo		Eriomin	
		0 Week	12° Week	0 Week	12° Week
Systolic blood pressure, mmHg		121 ± 13	123 ± 19	102 ± 25	102 ± 26
		*−0.7%*		*0.2%*	
Diastolic blood pressure, mmHg		76.0 ± 10.4	77.3 ± 8.7	76.0 ± 10.4	75.0 ± 11.1
		*1.8%*		*1.5%*	
Body weight, kg		99.6 ± 23.1	98.9 ± 23.1	102 ± 25	102 ± 26
		*−0.7%*		*0.0%*	
BMI, kg/m^2^		34.5 ± 6.5	34.5 ± 6.7	34.5 ± 7.1	34.5 ± 7.3
		*−0.3%*		*−0.2%*	
Lean mass, kg		34.5 ± 7.3	34.7 ± 7.4	34.9 ± 6.7	34.9 ± 7.3
		*0.5%*		*0.2%*	
Fat mass, kg		37.7 ± 8.9	38.3 ± 15.6	40.1 ± 17.8	39.3 ± 18.6
		*−1.6%*		*−1.8%*	
Ratio waist/hip		1.05 ± 0.10	1.05 ± 0.09	1.06 ± 0.08	1.06 ± 0.09
		*0.1%*		*0.6%*	

Two-way repeated measures ANOVA followed by Turkey test between groups (Placebo vs. Eriomin) over 12-wk intervention period.

**Table 6.** Dietary Parameters of Prediabetic Volunteers Submitted to Eriomin (200 mg/d) or Placebo for 12 Weeks

Variables		Placebo		Eriomin	
		0 Week	12° Week	0 Week	12° Week
Energy (kcal)		2619 ± 348	2560 ± 376	2596 ± 320	2634 ± 328
		*−2.2%*		*+1.6%*	
Carbohydrates (g)		336 ± 37	328 ± 59	344 ± 58	349 ± 57
		*−2.7%*		*+2.6%*	
Fibers (g)		19.0 ± 3.4	18.8 ± 3.0	19.3 ± 2.6	18.6 ± 3.6
		*0.002%*		*−3.0%*	
Protein (g)		137 ± 34	140 ± 36	142 ± 31	148 ± 33
		*+4.7%*		*+5.2%*	
Lipids (g)		144 ± 33	143 ± 39	145 ± 31	144 ± 37
		*−0.1%*		*−1.0%*	
Saturated fatty acids (g)		35.0 ± 14.0	34.1 ± 12.0	34.6 ± 9.0	34.9 ± 9.0
		*+2.9*		*+2.1%*	
Cholesterol (mg)		502 ± 213	516 ± 186	523 ± 188	534 ± 179
		*+8.5%*		*+2.8%*	
Vitamin C (mg)		55.7 ± 23.1	53.6 ± 23.0	56.3 ± 32.0	55.1 ± 29.5
		*−0.2%*		*−0.5%*	
Vitamin E (mg)		14.3 ± 2.0	14.6 ± 2.1	14.6 ± 2.1	14.5 ± 2.1
		*+2.3%*		*−0.3%*	

Two-way repeated measures ANOVA followed by Turkey test between groups (Placebo vs. Eriomin) over 12-wk intervention period.

*Italic values:* difference in percentage between week 12 and week 0.

## DISCUSSION

In this investigation, the effects of daily supplement with Eriomin for 12 weeks were evaluated in individuals with moderate to high levels of hyperglycemia (110–150 mg/dL), without complications and no use of hypoglycemic agents. A significant decrease in fasting glycemia (−5%), insulin resistance (−11%), ALP (−8%), glucagon (−13%), IL-6 (−14%), and TNF-*α* (−20%) was detected. In addition, levels of GLP-1 were significantly increased in the blood of the patients treated with Eriomin (+17%). Similar results were previously found with prediabetic patients taking 200, 400, and 800 mg/d of Eriomin for the same period.^12^ Although the two experiments are comparable, baseline fasting blood glucose levels are different between the two studies.

In the previous study,^12^ patients had hyperglycemia bordering on prediabetes (103 ± 13 mg/dL), while in the present study the mean hyperglycemia is higher (123 ± 18 mg/dL). This means that these patients were borderline for diabetes, and six of them already had hyperglycemia levels above 125 mg/dL, although they still did not use hypoglycemic drugs or were diagnosed with diabetes. Similarly, both study designs were double-blind, randomized, and placebo-controlled, but while the first study had two arms parallel-design (control and test), the second was a crossover, with two cycles of 12 weeks for each treatment, Placebo and Eriomin. Therefore, comparisons of the results have to be made with caution. Previously, after treatment with Eriomin, used as three multiple doses (200, 400, and 800 mg/d), 24% of the hyperglycemic patients reverted to normal levels and/or lower glucose intolerance.^12^

However, in the present study, only three patients from both treatments (Eriomin or Placebo) reverted to normal levels of glycemia. Therefore, the active supplement (Eriomin) cannot be pointed as the causal factor for this reversion.

Under normal physiological conditions, intestinal L cells secrete an incretin, known as GLP-1, shortly after carbohydrate ingestion. This GLP-1 will act in the regulation of glucose metabolism, stimulating insulin secretion, while reducing glucagon secretion, thus reducing circulating glucose.^15^ However, in the prediabetic state, GLP-1 secretion is decreased, which impairs blood glucose control.^16^ Therefore, the detected 17% increase in GLP-1 in the blood serum of patients after treatment with Eriomin, associated with a 13% reduction in glucagon levels are pointed out as the likely causes of the decrease in hyperglycemia observed in this study.

These results were very similar to a previous study,^12^ in which 200 mg/d of Eriomin increased 15.6% the levels of GLP-1 and decreased 6% the glucagon in the blood. Although both studies showed a similar effect of Eriomin on GLP-1, the glucagon reduction was twofold greater in the current study, presumably due to higher baseline levels of hyperglycemia.

The glucose-lowering potential of polyphenols has been evidenced in several acute and chronic models, and in healthy and diabetic organisms, showing effects similar to hypoglycemic drugs, and therefore they are potential candidates to control glucose homeostasis and act as a medicine alternative to T2D.^17^ Previously, we have shown, in mice fed high-fat-diet and supplemented with eriocitrin, a significant decrease in glucose and triacylglycerol and improvement of insulin sensitivity, associated with less oxidative stress and systemic inflammation, promoted by this citrus flavonoid.^18^

Increased production of proinflammatory cytokines also plays an important role in the pathogenesis of T2D, and contributes to long-term micro- and macrovascular complications.^19,20^ In the course of prediabetes, it is common for patients to have higher levels of inflammatory markers, such as IL-6 and TNF-*α*,^21^ observed in the current and in the previous study.^12^ In contrast, supplementation with Eriomin led to downregulation of low-grade inflammation in these patients, mainly due to reduced serum levels of these markers: IL-6 (−14%) and TNF-*α* (−20%). This result is similar to the previous study by our group, where patients also showed downregulation of inflammatory markers, even with lower hyperglycemia.^12^ In another study with mice, supplementation with Eriodictiol, the aglycone form of Eriocitrin that is the main component of Eriomin, also showed inhibitory effects on the expression of IL-6 and TNF-*α* messenger RNA.^22^

Others also found decreased IL-6 levels in mice fed a high-fat diet supplemented with eriocitrin or eriodictyol.^23^ The anti-inflammatory properties of these compounds result from the activation of peroxisome proliferator- activated receptor gamma (PPAR*γ*) expression and inhibition of nuclear factor kappa B, with consequent reductions in the secretion of inflammatory cytokines and increased adiponectin.^24,25^

As it was mentioned before, Eriomin regular supplementation is well tolerated by patients, since they did not report any discomfort or inability to continue to take the supplement. In addition, liver enzymes AST, ALT, and *γ*GT levels remained at normal levels, and ALP significantly decreased 8%, suggesting no hepatotoxicity of Eriomin at a dose of 200 mg/d, or even of higher doses, such as 400 and 800 mg/d tested before.^12,^^25^ A mice study showed that eriocitrin, the main flavonoid of Eriomin, protected against liver damage caused by regular consumption of a high-fat diet.^23^ Similarly, no changes were detected in the levels of urea and creatinine, thus showing normal kidney function with Eriomin during the experiment.

**FIG. 2.**
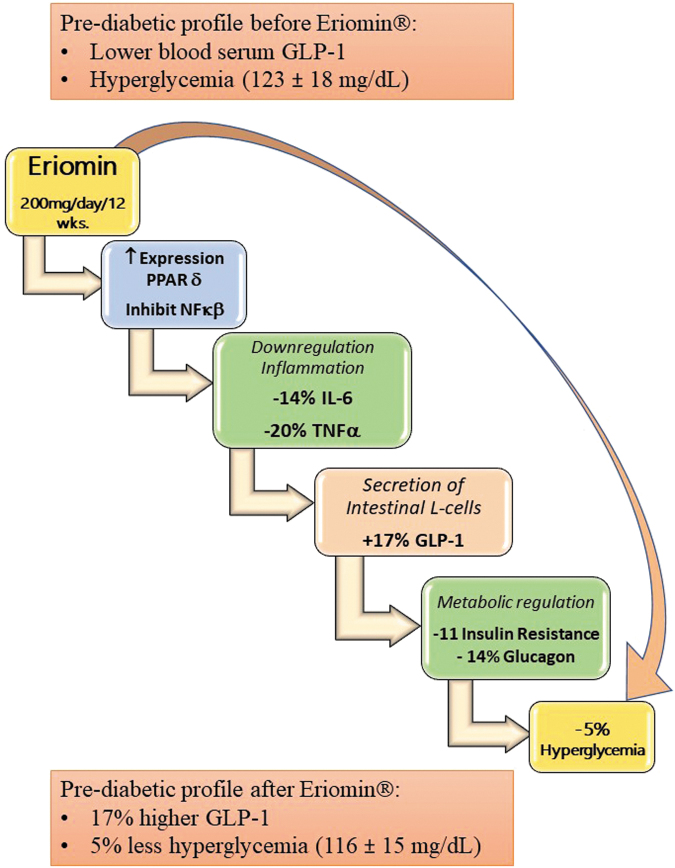
Event sequence after treatment with Eriomin for 12 weeks in prediabetic patients with moderate to high levels of hyperglycemia (110 to 150 mg/dL). GLP-1, glucagon-like peptide 1; IL-6, interleukin 6; NF*κ*B, nuclear factor kappa B; PPAR*γ*, peroxisome proliferator- activated receptor gamma; TNF-*α*, tumor necrosis factor alpha.

In conclusion, this study showed that 12 weeks of Eriomin supplementation, at 200 mg/d, resulted in a reduction in hyperglycemia (−5%), and a diminished inflammatory response, by decreasing blood serum markers IL-6 (−14%) and TNF-*α* (−20%). Simultaneously to the downregulation of inflammation, an increase in GLP-1 secretion (17%) and a decrease in Glucagon (−13%) were observed. Therefore, Eriomin was able to reduce hyperglycemia by increasing or promoting GLP-1 secretion, benefiting patients with moderate to high levels of hyperglycemia. Figure 2 shows a sequence of these events, before and after Eriomin supplementation.

## Notes

^*^
Correction added on July 25, 2022, after first online publication of July 7, 2022: The Clinical Trial Registration number has been corrected from (NCT03215043) to (NCT03928249).
